# Kardiovaskuläre Komplikationen unter Androgenentzugstherapie: Vorteil für Gonadotropin-Releasing-Hormon-Antagonisten? Ein Update

**DOI:** 10.1007/s00120-021-01583-9

**Published:** 2021-07-02

**Authors:** Gunhild von Amsberg, Holger Thiele, Axel Merseburger

**Affiliations:** 1 II. medizinische Klinik, Onkologisches Zentrum und Martini-Klinik, Universitätsklinik Hamburg-Eppendorf, Martinistr. 52, 20246 Hamburg, Deutschland; 2 Herzzentrum Leipzig, Universitätsklinik für Kardiologie, Leipzig, Deutschland; 3 Klinik für Urologie, Universitätsklinikum Schleswig-Holstein, Lübeck, Deutschland

**Keywords:** Prostatakarzinom, Relugulix, HERO-Studie, Degarelix, STAMP, Prostate cancer, Relugolix, HERO trial, Degarelix, STAMP

## Abstract

**Hintergrund:**

Die Androgendeprivationstherapie (ADT) spielt in der Behandlung des fortgeschrittenen Prostatakarzinoms eine zentrale Rolle. Der zusätzliche Einsatz neuer Medikamente führt sowohl in der hormonsensitiven Situation als auch der Kastrationsresistenz zu einem verlängerten Gesamtüberleben. Ein dadurch bedingter, langjähriger Einsatz der ADT rückt mögliche Komplikationen in den Vordergrund. Dies gilt insbesondere für kardiovaskuläre Ereignisse.

**Ziel der Arbeit:**

Das Ziel der Arbeit war die Prüfung der aktuellen Datenlage zu möglichen Unterschieden des kardiovaskulären Risikoprofils von Gonadotropin-Releasing-Hormon- (GnRH-)Agonisten und GnRH-Antagonisten.

**Methoden:**

Narrativer Bericht basierend auf einem Expertenkonsens, unterstützt von einer Literaturrecherche in PubMed (MEDLINE) und den Abstract Datenbanken von ASCO und ESMO zwischen Januar 2015 und 2021. Berücksichtigt wurden für den Behandlungsalltag bedeutsame Metaanalysen, vergleichenden randomisierte klinische Studien (RCT) und „real world data“ (RWD). Die Studienauswahl wurde hinsichtlich der klinischen Relevanz für den Praxisalltag vorgenommen.

**Ergebnisse:**

Es wurden drei für die Thematik relevante Metaanalysen, zwei prospektive RCT sowie drei RWD-Publikationen identifiziert. Dabei zeigt sich übereinstimmend ein Vorteil für GnRH-Antagonisten mit einer geringeren Inzidenz kardiovaskulärer Ereignisse im Vergleich zu GnRH-Agonisten. Lediglich eine RWD-Untersuchung berichtet über eine vergleichbare Komplikationsrate mit beiden Substanzgruppen.

**Schlussfolgerung:**

Die GnRH-Antagonisten weisen ein geringeres Risiko für das Auftreten kardiovaskulärer Ereignisse als GnRH-Agonisten auf. Eine Risikominimierung sollte durch Berücksichtigung bekannter kardiovaskulärer Risikofaktoren vor Therapieeinleitung vorgenommen werden.

## Abstract (en)

**Background:**

Androgen deprivation therapy (ADT) plays a crucial role in treatment of advanced prostate cancer (PCa). The additional application of new drugs results in prolonged overall survival, both in the hormone sensitive and castration resistant state. Consequently, the long-term use of ADT moves potential side effects into the focus of interest. In this context special consideration must be given to cardiovascular events.

**Objectives:**

Review of current evidence on potential differences regarding the cardiovascular risk profile of gonadotropin-releasing hormone (GnRH) agonists compared to GnRH antagonists.

**Methods:**

Narrative review based on an expert consensus supported by a literature search in PubMed (MEDLINE) and the abstract databases of ASCO and ESMO was conducted for publications published between January 2015 and January 2021. Significant meta-analyses, randomized controlled trials (RCTs) and real-world data (RWD) revealing relevant results for clinical practice were taken into account. Selection of studies was performed based on the clinical relevance for everyday practice.

**Results:**

The search yielded three relevant meta-analyses, two prospective RCTs as well as three RWD publications that are of importance for clinical practice. Overall, a decreased incidence of cardiovascular events was reported for GnRH antagonists compared to GnRH agonists. Only one RWD publication described comparable rates of complications for both drug classes.

**Conclusion:**

GnRH antagonists have a lower risk of treatment related cardiovascular events compared to GnRH agonists. Risks should be minimized by taking known cardiovascular risk factors into account before initiating therapy.

Seit unserer ersten Publikation „Kardiovaskuläre Risikopatienten unter Androgenentzugstherapie: Geringeres Risiko mit Gonadotropin-Releasing-Hormon- (GnRH-)Antagonisten im Vergleich zu GnRH-Agonisten?“ im Jahr 2016 [27] hat sich die Behandlung des fortgeschrittenen Prostatakarzinoms (PCa) grundlegend verändert. Dies gilt insbesondere für das hormonsensitive Stadium. Unverändert zentraler Therapiebestandteil bleibt die Androgendeprivationstherapie (ADT). Anlass für uns nun, 5 Jahre später, die kardiovaskulären Risiken der ADT erneut ins Visier zu nehmen.

## Hintergrund

Seit vor 80 Jahren Huggins und Hodges erstmalig den Nachweis erbrachten, dass das Wachstum des PCa Androgen-abhängig erfolgt, ist der Androgenentzug zentraler Bestandteil der Therapie des fortgeschrittenen PCa. Tatsächlich war die alleinige Therapie mit Gonadotropin-Releasing-Hormon- (GnRH-)Agonisten oder -Antagonisten lange Standard im hormonsensitiven Stadium mit einem klinischen und biochemischen Ansprechen von > 90 % und einem progressionsfreien Überleben von ca. 18 bis 24 Monaten. Zu einem Paradigmenwechsel führten die Ergebnisse der CHAARTED-Studie mit einem signifikanten Überlebensvorteil durch die Hinzunahme von Docetaxel, insbesondere bei Patienten mit hoher Tumorlast [38]. Seither haben sich die Ereignisse förmlich überschlagen. Intensivierte Hormontherapien mit neuen Androgenrezeptor- (AR-)gerichteten Medikamenten („AR targeting agent“, ARTA) zeigten in Phase-III-Studien vergleichbare Aktivitäten wie die Hormonchemotherapie, allerdings mit einer vorteilhafteren Akuttoxizität. Es folgten die Zulassung von Abirateron/Prednisolon für Patienten mit hohem Risikoprofil und de novo metastasiertem PCa sowie Apalutamid im Frühjahr 2020 für eine „All-comer-Population“, unabhängig von Metastasenlast, Gleason-Score und lokaler Vortherapie [2, 14]. Eine ähnliche Zulassungserweiterung wird für Enzalutamid erwartet [3, 11].

Auch bei Progress der Erkrankung mit einem Übergang in das kastrationsresistente Stadium gab es Neuerungen. So wurde jüngst der Poly-(ADP-Ribose‑)Polymerase- (PARP-)Inhibitor Olaparib für Patienten mit Nachweis einer *BRCA1/2*-Mutation nach Vorbehandlung mit einem ARTA basierend auf den Ergebnissen der PROFOUND-Studie zugelassen [16]. Für Patienten mit kastrationsresistentem PCa ohne Nachweis einer Metastasierung und kurzer PSA-Verdopplungszeit (prostataspezifisches Antigen) < 10 Monaten stehen mit Apalutamid, Darolutamid und Enzalutamid zudem drei Substanzen zur Verfügung, die in Phase-III-Studien zu einer signifikanten Verlängerung des metastasenfreien Überlebens und des Gesamtüberlebens geführt haben [12, 13, 15, 34–36].

Dabei werden die verschiedenen Behandlungsoptionen stadienunabhängig mit einer medikamentösen ADT kombiniert. Weitere Einsatzbereiche der ADT sind die neoadjuvante oder adjuvante Therapie begleitend zur Strahlentherapie oder auch das biochemische Rezidiv. Damit erfolgt die ADT häufig während der gesamten Erkrankungsdauer und begleitet die Patienten über viele Jahre. Dies könnte auch ursächlich dafür sein, warum Komplikationen der ADT im Behandlungsalltag häufig nur unzureichend erfasst werden. So dürfte die ADT nicht immer als möglicher Auslöser für einen unerwünschten Vorfall identifiziert werden. Zudem ist anzunehmen, dass es auch von Seiten der Patienten zu einem gewissen „under-reporting“ kommt. Obwohl sich die Nebenwirkungen von GnRH-Agonisten und -Antagonisten vielfach ähneln, scheint es auch relevante Unterschiede zu geben. Tatsächlich mehren sich die Hinweise, dass GnRH-Antagonisten einen klinisch bedeutsamen Vorteil hinsichtlich kardiovaskulärer Komplikationen im Vergleich zu GnRH-Agonisten aufweisen [27]. Im Rahmen dieser Übersichtsarbeit werden aktuelle Studienergebnisse zu dieser Thematik zusammengefasst und für den Behandlungsalltag ausgewertet.

## Neue Evidenz zeigt geringeres kardiovaskuläres Risiko unter GnRH-Antagonisten gegenüber GnRH-Agonisten

Im Rahmen dieser Übersichtsarbeit wurden praxisrelevante Publikationen ausgewählt, die zwischen dem 01. Januar 2015 und dem 31. Januar 2021 in Form von Vollpublikationen oder Abstracts veröffentlicht wurden und die das Auftreten kardiovaskulärer Ereignisse unter GnRH-Agonisten oder GnRH-Antagonisten oder beiden Substanzklassen untersuchten. Die Ergebnisse sind in Tab. 1 zusammengefasst und werden im Folgenden ausführlich dargestellt.

**Table 1:** 

Autor	Jahr	Studientyp	Ergebnis bzgl. Risiko für das Auftreten kardiovaskulärer Ereignisse	Referenz
Cirne	2021	MA	Vorteil für GnRH-Antagonist	[7]
Abufaraj	2020	MA	Vorteil für GnRH-Antagonist	[1]
Bosco	2015	MA	Erhöhtes Risiko für GnRH-Agonisten	[5]
Margel	2019/ 2020	RCT	Vorteil für GnRH-Antagonist	[25, 26]
Shore	2020	RCT	Vorteil für GnRH-Antagonist	[33]
Cardwell	2020	RWD	Erhöhtes Risiko für GnRH-Agonisten und -Antagonisten	[6]
Davey	2020	RWD	Vorteil für GnRH-Antagonist	[10]
Perrone	2020	RWD	Vorteil für GnRH-Antagonist	[30]

*MA* Metaanalyse, *RCT* randomisierte kontrollierte Studie, *RWD* „real world data“, *GnRH* Gonadotropin-Releasing-Hormon

### Prospektiv randomisierte Studien zeigen einen Vorteil für GnRH-Antagonisten

In den vergangenen 5 Jahren erschienen relevante Ergebnisse zweier RCT zur Thematik kardiovaskulärer Ereignisse unter ADT [26, 33].

Für besonderes Aufsehen sorgte dabei die Phase-III-Studie HERO, die auf dem virtuellen Kongress der American Society of Clinical Oncology (ASCO) 2020 präsentiert wurde [33].

Hier wurde der neu entwickelte orale GnRH-Antagonist Relugolix mit dem GnRH-Agonisten Leuprorelin bei Patienten mit biochemischem Rezidiv oder fortgeschrittenen PCa verglichen. Die Studie erreichte ihren primären Endpunkt mit einer anhaltenden Kastration bei 96,7 % der Relugolix-Patienten im Vergleich zu 88,8 % der Patienten unter Leuprorelin über einen Zeitraum von 48 Wochen. Überlegen war der GnRH-Antagonist dem GnRH-Agonisten sowohl bezüglich des PSA-Ansprechens mit 79,4 % vs. 19,8 % als auch hinsichtlich der kumulativen Wahrscheinlichkeit einer Testosteronsuppression < 50 ng/dl bzw. < 20 ng/dl an Tag 15 (98,71 vs. 12,05 % und 78,38 vs. 0,98 %; jeweils *p* < 0,0001). Von besonderer Bedeutung für den Behandlungsalltag dürfte jedoch insbesondere die um 54 % geringere Rate kardiovaskulärer Ereignisse unter Therapie mit GnRH-Antagonisten sein. So betrug die Inzidenz schwerwiegender kardiovaskulärer Ereignisse nach 48 Wochen 2,9 % in der Relugolix-Gruppe im Vergleich zu 6,2 % in der Leuprorelin-Gruppe (Hazard Ratio [HR]: 0,46; 95%-Konfidenzintervall [‑KI]: 0,24–0,88; [33]). Diese umfassten nicht tödlich verlaufende Myokardinfarkte oder Schlaganfälle sowie Todesursachen jeglicher Genese. Bei Patienten mit schwerwiegenden kardiovaskulären Ereignissen in der Anamnese erhöhte sich das Risiko eines erneuten Vorfalls in der Leuprorelin-Gruppe um das 4,8-Fache im Vergleich zu Relugolix (17,8 % vs. 3,6 %). Besonders hervorzuheben ist, dass bei Studieneinschluss > 90 % der Patienten kardiovaskuläre Risikofaktoren aufwiesen [33]. Relugolix wurde im Dezember des vergangenen Jahres durch die Food and Drug Administration (FDA) zugelassen, die Zulassung bei der Europäische Arzneimittelagentur (EMA) wurde im März dieses Jahres beantragt [29].

Eine randomisierte, offene Phase-II-Studie bei PCa-Patienten mit vorbestehender kardiovaskulärer Erkrankung untersuchte die Endothelfunktion sowie das Auftreten eines erneuten kardiovaskulären Ereignisses in Abhängigkeit einer Therapie mit GnRH-Agonisten (*n* = 39) oder -Antagonisten (*n* = 41; [26]). Hinsichtlich der Endothelfunktion konnte kein statistisch signifikanter Unterschied festgestellt werden. Allerdings traten bei 15 Studienteilnehmern neue kardiovaskuläre Ereignisse im Rahmen der Studie auf, die bei 9 Patienten als schwerwiegend eingestuft wurden (Tod: *n* = 2; Myokardinfarkt: *n* = 1; zerebrovaskuläre Ereignisse: *n* = 2; perkutane Koronarintervention mit Anlage eines Koronarstents: *n* = 4). Bemerkenswert war, dass 20 % der Patienten mit einem GnRH-Agonisten, aber nur bei 3 % der Patienten mit GnRH-Antagonisten betroffen waren (*p* = 0,013). Somit führte der Einsatz des GnRH-Antagonisten zu einer absoluten Risikoreduktion um 18,1 % (95%-KI: 4,6–31,2; *p* = 0,032) für schwerwiegende kardio- und zerebrovaskuläre Ereignisse nach 12 Monaten [26].

Beide Studien zeigen somit einen Vorteil für GnRH-Antagonisten gegenüber GnRH-Agonisten bezüglich der Inzidenz kardiovaskulärer Ereignisse unter der ADT. Weitere Daten zum kardiovaskulären Nebenwirkungsspektrum von GnRH-Agonisten und -Antagonisten wird die PRONOUNCE-Studie (NCT02663908) liefern. In dieser globalen, randomisierten Phase-III-Studie wird die Inzidenz schwerwiegender kardiovaskulärer Ereignisse erstmalig als primärer Studienendpunkt bei Patienten mit entsprechenden Vorerkrankungen unter Therapie mit Degarelix oder Leuprorelin verglichen.

### Metaanalysen implizieren geringeres kardiovaskuläres Risiko von GnRH-Antagonisten

Eine aktuelle systemische Metaanalyse von Cirne et al. vom Januar dieses Jahres analysierte insgesamt 10 Studien bezüglich der Inzidenz kardiovaskulärer Ereignisse unter Therapie mit den GnRH-Antagonisten Degarelix (*n* = 1681) und Relugolix (*n* = 734) im Vergleich zu den GnRH-Agonisten Leuprorelin (*n* = 714) und Goserelin (*n* = 600; [7]). Dabei zeigte sich in der zusammenfassenden Auswertung ein Vorteil für GnRH-Antagonisten mit einem 43 % geringeren Risiko für kardiovaskuläre Ereignisse sowie einem 51 % bzw. 52 % geringeren Risiko an einem kardiovaskulären Ereignis zu versterben bzw. einem Tod jeglicher Genese. Die Ergebnisse der Metaanalyse wurden in Tab. 2 zusammengefasst.

**Table 2:** 

	GnRH-Antagonisten *n* = 2415	GnRH-Agonisten *n* = 1345	Gepooltes RR (95 %-KI)
Kardiovaskuläre Ereignisse (%)	3,5	6,5	0,57 (0,39–0,81)
Kardiovaskulär bedingte Todesfälle (%)	0,8	1,6	0,49 (0,25–0,96)
Todesfälle jeglicher Genese (%)	1,0	2,3	0,48 (0,28–0,83)

*GnRH* Gonadotropin-Releasing-Hormon, *KI* Konfidenzintervall, *RR* relatives Risiko

Besonders hervorgehoben wurde von den Autoren, dass der Vorteil für beide GnRH-Antagonisten beobachtet wurde und somit ein Klasseneffekt sein könnte. Sie schlussfolgerten, dass eine Blockade des GnRH-Rezeptors im Gegensatz zu einer anhaltenden Stimulation des GnRH-Rezeptors mit einer protektiven Wirkung für die Entstehung kardiovaskulärer Erkrankungen einhergehen könnte.

Die Metaanalyse von Abufaraj et al. vom Juni des vergangenen Jahres untersuchte mögliche Unterschiede zwischen GnRH-Agonisten und GnRH-Antagonisten bezüglich klinischer Verträglichkeit und onkologischer Wirksamkeit [1]. Ausgewertet wurden 8 prospektiv randomisierte Studien mit insgesamt 2632 mPCa-Patienten. Davon erhielten 1646 Patienten einen GnRH-Antagonisten (Degarelix) und 986 Patienten einen GnRH-Agonisten. Auch hier waren GnRH-Antagonisten mit statistisch signifikant weniger kardiovaskulären Ereignissen assoziiert (relatives Risiko [RR]: 0,52; 95%-KI: 0,34–0,80; *p* = 0,003). Während kein signifikanter Unterschied in der PSA-Progression bestand, wurde unter GnRH-Antagonisten eine geringere Gesamtmortalität als unter GnRH-Agonisten verzeichnet (RR: 0,48; 95%-KI: 0,26–0,90; *p* = 0,02; [1]).

Eine ältere Metaanalyse von Bosco et al. aus 2015 fokussierte sich auf die Auswirkungen des Androgenentzugs mit GnRH-Agonisten auf das Risiko kardiovaskulärer Ereignisse im Vergleich zu PCa-Patienten, bei denen keine ADT durchgeführt wurde. Hierzu erfolgte die Analyse von 8 Beobachtungsstudien [5]. Auch hier waren GnRH-Agonisten wieder mit einem erhöhten kardiovaskulären Risiko assoziiert. So betrug das relative Risiko für das Auftreten einer nicht-fatalen kardiovaskulären Erkrankung jeglicher Art 1,38 (95%-KI: 1,29–1,48) im Vergleich zur Kontrollgruppe, das RR für eine nicht-tödlich verlaufende ischämische Herzerkrankung 1,39 (95%-KI: 1,26–1,54). Noch deutlicher war der Unterschied für nicht-tödlich oder tödlich verlaufende Myokardinfarkte (RR: 1,57; 95%-KI: 1,26–1,94) oder Schlaganfälle (RR: 1,51; 95%-KI: 1,24–1,84; [5]). GnRH-Antagonisten wurden in dieser Metaanalyse nicht berücksichtigt.

Für eine systematische Übersicht der Evidenzlage zur Anwendung des GnRH-Antagonisten Degarelix wird auf den entsprechenden Cochrane-Review verwiesen, dessen Publikation in Kürze erwartet wird [18].

### Daten aus dem Behandlungsalltag bestätigen den Vorteil von GnRH-Antagonisten

Verschiedene Beobachtungsstudien bestätigen die zuvor berichteten Vorteile von GnRH-Antagonisten im Vergleich zu GnRH-Agonisten hinsichtlich der kardiovaskulären Toxizität.

In einer retrospektiven Beobachtungsstudie aus Italien [30] erhielten von 9785 Patienten mit PCa 93,6 % einen GnRH-Agonisten und 6,4 % Degarelix. Dabei war die Inzidenz kardiovaskulärer Ereignisse statistisch signifikant höher bei Patienten, die mit GnRH-Agonisten anstelle von Degarelix behandelt wurden (8,8 vs. 6,2 Ereignisse pro 100 Personenjahre, *p* = 0,002). Die multivariate Regressionsanalyse bestätigte ein signifikant geringeres Risiko für das Auftreten eines kardiovaskulären Ereignisses unter Degarelix-Therapie (HR 0,76; 95%-KI: 0,60–0,95, *p* = 0,018). Dies traf auch auf die Subgruppe der Patienten ohne vorherige kardiovaskuläre Ereignisse zu [30].

Auch eine RWD-Analyse aus der britischen Primärversorgung zeigte einen Vorteil des GnRH-Antagonisten Degarelix im Vergleich zu GnRH-Agonisten mit einer geringeren Inzidenz kardialer Ereignisse (Risikoverhältnis: 0,39; 95%-KI: 0,191–0,799; *p* = 0,01; [10]). Ausgewertet wurden hierzu 101 Patienten unter Degarelix-Therapie sowie 3289 Leuprorelin-, 4366 Goserelin- und 1325 Triptorelin-Patienten [10].

Eine länderübergreifende Auswertung wurde auf dem ASCO 2020 präsentiert. Hier wurden Daten der World Health Organization (WHO) aus über 130 Ländern zu kardialen Ereignissen unter Degarelix-Behandlung mit den GnRH-Agonisten Leuprolid, Goserelin, Triptorelin oder Histrelin verglichen [8]. In Übereinstimmung mit den anderen Studien wiesen GnRH-Agonisten ein signifikant höheres Risiko für kardiovaskuläre Ereignisse als GnRH-Antagonisten auf (Odds Ratio [OR]: 1,20; 95%-KI: 1,12–1,29). Dies galt insbesondere auch für Myokardinfarkte (OR: 1,76; 95%-KI: 1,57–1,97) und Herzinsuffizienz (OR: 2,02; 95%-KI: 1,73–2,35) [8].

Eine einzige Analyse von Cardwell et al. aus Schottland mit 20.216 PCa-Patienten zeigte ein gleichermaßen erhöhtes kardiovaskuläres Risiko für eine ADT mit GnRH-Agonisten und Degarelix um 30 % (adjustierte HR: 1,3; 95%-KI: 1,2–1,4; [6]). Dies traf dagegen nicht auf die Bicalutamid-Monotherapie zu (adjustierte HR: 1,0; 95%-KI: 0,82–1,3; [6]). Dabei wiesen die Autoren darauf hin, dass dies möglicherweise auf eine Verzerrung durch die Patientenauswahl innerhalb der Studie zurückgeführt werden könnte. So wurden mehr Patienten in fortgeschrittenem Stadium mit dem GnRH-Antagonisten behandelt und hatten damit möglicherweise ein erhöhtes tumorbedingtes Risiko für ein kardiovaskuläres Ereignis.

### Tumorerkrankung und Lebensstil als zusätzliches Risiko für kardiovaskuläre Ereignisse

Ein erhöhtes kardiovaskuläres Risiko für PCa-Patienten wurde auch im Vergleich zur Normalbevölkerung nachgewiesen. So zeigte sich in einer bevölkerungsbasierten Studie in den USA mit über 3 Mio. Tumorpatienten, dass mehr als jeder 10. Patient an einem kardiovaskulären Ereignis verstarb [37]. Am häufigsten waren Patienten mit Brust‑, Prostata- oder Blasenkrebs betroffen [37]. Die kanadische RADICAL-PC-Studie mit ca. 2500 Patienten kam zu dem Schluss, dass ca. 70 % der Patienten mit PCa ein hohes kardiovaskuläres Risiko basierend auf den Kriterien des Framingham-Risikoscores aufweisen [21], d. h. ein mindestens 20%iges Risiko besitzen, innerhalb von 10 Jahren ein kardiovaskuläres Ereignis zu erleiden. Selbst bei den hochselektionierten Patienten der Martini-Lifestyle-Kohorte war die Adhärenz zu den Empfehlungen des World Cancer Research Funds (WCRF) und dem American Institute of Cancer Research (AICR) gering.

Tatsächlich wiesen zwei Drittel der Patienten kein Normalgewicht auf und ca. 50 % der Patienten rauchten [39].

Diese Daten verdeutlichen, warum es unerlässlich ist, vor Therapieeinleitung eine sorgfältige Anamnese durchzuführen, Risikofaktoren zu erfassen und im Hinblick auf das Nebenwirkungsprofil der geplanten Medikation zu berücksichtigen. Dabei sollten mögliche Patienten- oder Behandlungsbedingte Risikofaktoren soweit wie möglich reduziert werden. Eine enge interdisziplinäre Zusammenarbeit mit den kardiologischen Kollegen, bevorzugt mit Anbindung an eine kardioonkologische Ambulanz oder spezialisierte Klinik ist wünschenswert (Tab. 3).

**Table 3:** 

Das Wichtigste auf einen Blick
Unter ADT ist das Risiko kardiovaskulärer Ereignisse erhöht
**Erhöhtes kardiovaskuläres Risiko/kardiovaskuläre Vorgeschichte?**
*Identifizieren*: Vorbestehende Erkrankung: STAMP Klassische Risikofaktoren: Arterielle Hypertonie, Rauchen, Hyperlipidämie, Alter, Diabetes, Übergewicht *Behandeln*: Optimierung der medikamentösen Einstellung in Zusammenarbeit mit Kardiologen Hinweise zum Lebensstil *Überwachen*: Abfragen von Symptomen kardiovaskulärer Erkrankungen Messung des Blutdrucks Laborwerte bestimmen (LDL, HbA1c)
**Daran denken:**
Frühzeitige kardiologische Mitbetreuung Möglicher Vorteil von GnRH-Antagonisten gegenüber -Agonisten bezgl. kardiovaskulärer Ereignisse Erhöhtes kardiovaskuläres Risiko beim gelichzeitigen Einsatz neuer AR-gerichteter Medikamente

*ADT* Androgendeprivationstherapie, *GnRH* Gonadotropin-Releasing-Hormon, *LDL* „low density lipoprotein“, *AR* Androgenrezeptor

### Erhöhtes kardiovaskuläres Risiko durch Einsatz neuer AR-gerichteter Therapien

Therapien mit den neuen AR-gerichteten Substanzen Abirateron, Apalutamid, Darolutamid und Enzalutamid scheinen zudem das Risiko kardiovaskulärer Ereignisse zu erhöhen. Dabei können allerdings basierend auf den Zulassungsstudien nur eingeschränkt Aussagen zu Patienten mit schwerwiegenden Vorerkrankungen des Herz-Kreislauf-Systems getroffen werden, da dies häufig Ausschlusskriterium für eine Studienteilnahme war. Allerdings zeigte sich in einer aktuellen retrospektiven Analyse von 2845 Patienten unter Abirateron/Prednisolon- und 1031 Patienten unter Enzalutamid-Therapie eine Steigerung des 6‑Monate-Mortalitätsrisiko um 16 %, wenn die Patienten ein bis zwei kardiovaskulären Vorerkrankungen aufwiesen (RR: 1,16; 95%-KI: 1,00–1,88; [23]). Dieses Risiko stieg mit einer wachsenden Zahl kardiovaskulärer Vorerkrankungen weiter an. Ein signifikanter Unterschied zwischen den beiden ARTA wurde dabei nicht festgestellt. Alarmierend ist, dass in dieser populationsbasierten Analyse ca. zwei Drittel der Patienten an mindestens einer kardiovaskulären Vorerkrankung litten und somit ein erhöhtes Risiko aufwiesen. Eine Metaanalyse von 7 prospektiven Studien mit insgesamt 8660 Patienten brachte die neuen ARTA ebenfalls mit einem signifikant erhöhten Risiko für das Auftreten kardialer Ereignisse jeglichen Grades (RR: 1,36; 95%-KI: 1,13–1,64; *p* = 0,001) und schwerer Ausprägung in Verbindung (RR: 1,84; 95%-KI: 1,21–2,80; *p* = 0,004; [17]). Zudem wurden signifikant häufiger Blutdruckerhöhungen jeglicher und hochgradiger Ausprägung im Vergleich zu den Kontrollpatienten beobachtet (RR: 1,98; 95%-KI: 1,62–2,43; *p* = 0,001 vs. RR: 2,26; 95%-KI: 1,84–2,77; *p* = 0,004).

## Ausblick

Die hohe Koinzidenz von PCa und kardiovaskulären Erkrankungen ist aufgrund der hohen Prävalenz beider Krankheitsbilder nicht überraschend. Zudem begünstigen ähnliche Risikofaktoren wie beispielsweise ein fortgeschrittenes Patientenalter, das metabolische Syndrom, eine geringe körperliche Aktivität sowie eine fortschreitende Organverfettung das Auftreten der beiden Erkrankungen. ADT und neue AR-gerichtete Medikamente erhöhen das Risiko für kardiovaskuläre Ereignisse zusätzlich. Tatsächlich hat mittlerweile die kardiovaskuläre Mortalität die tumorspezifische Sterblichkeit als häufigste Todesursache bei PCa-Patienten abgelöst [28].

Dennoch ist der Aspekt der kardiovaskulären Nebenwirkungen in der S3-Leitlinie zur Behandlung des PCa bislang nicht abgebildet [20]. Dagegen wurde von der American Urological Association (AUA) bereits im Jahr 2010 durch einen interdisziplinären Übersichtsartikel in Zusammenarbeit mit der American Heart Association (AHA) und der American Cancer Association (ACA) auf dieses Thema aufmerksam gemacht [22]. Dies verdeutlicht, warum wir in unserer täglichen Praxis ein noch stärkeres Augenmerk auf die Erfassung und das Monitoring von kardiovaskulären Risikofaktoren legen sollten.

**Figure 1:**
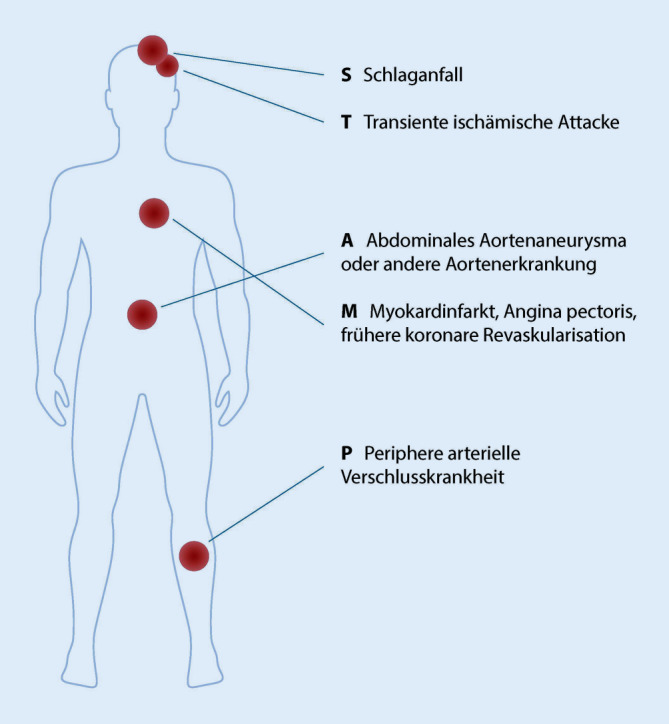

Dieser Herausforderung hat sich eine multidisziplinäre, kanadische Arbeitsgruppe angenommen und ein einfaches, praxistaugliches Screeningtools (STAMP) zur Identifizierung vorbestehender kardiovaskulärer Erkrankungen entwickelt (Abb. 1, [19]): Im Rahmen des Screenings mit „STAMP“ werden Vorerkrankungen wie Schlaganfall, transiente ischämische Attacke, abdominelles Aortenaneurysma bzw. andere Aortenerkrankungen, Myokardinfarkt, Angina pectoris oder frühere koronare Revaskularisation sowie die periphere arterielle Verschlusskrankheit systematisch erfasst. Weist ein Patient eine oder mehrere dieser Erkrankungen auf, empfehlen die Autoren die Einleitung medikamentöser Maßnahmen, die je nach Art und Schwere der kardiovaskulären Vorerkrankung die Gabe von Acetylsalicylsäure, „Angiotensin converting enzyme“- (ACE-)Hemmer oder Angiotensin-1-Antagonisten, sowie ADP-Rezeptorinhibitoren und Statinen als Lipidsenker umfassen können. Insbesondere bei den letzten beiden Medikamentengruppen muss allerdings auf mögliche Wechselwirkungen mit den jeweiligen Tumortherapien geachtet werden. So wurden gerade mit den neuen ARTA relevante Medikamenteninteraktionen nachgewiesen, die es vor Therapieeinleitung zu prüfen gilt. Aus unserer Sicht ist daher eine enge Zusammenarbeit zwischen Uroonkologen und Kardiologen unerlässlich.

Unsere Aufgabe als Behandler ist es, die Patienten umfassend über mögliche kardiovaskuläre Komplikationen zu informieren und Maßnahmen zur Risikominimierung anzustoßen. Dazu gehören neben medikamentösen Interventionen wie beispielweise der Blutdruckeinstellung und Optimierung einer Diabetesbehandlung, auch die Männer zu einem aktiven Lebensstil, einer Ernährungsumstellung, sportlichen Betätigung oder einer Nikotinentwöhnung zu ermutigen [19, 31]. In der Praxis sollten Symptome kardiovaskulärer Erkrankungen regelmäßig abgefragt, der Blutdruck gemessen (Zielblutdruck 140/90 mm Hg für alle, bei guter Tolerierung Zielblutdruck 130/80 mm Hg; für Patienten ≥ 65 Jahre Zielblutdruck systolisch zwischen 130–139 mm Hg [40]) und entsprechende Laborwerte bestimmt werden (Ziel-LDL-Cholesterin risikoadaptiert 2,6 mmol/l, hohes Risiko < 1,8 mmol/l, sehr hohes Risiko < 1,4 mmol/l [24]). Außerdem sollte eine Diabetestherapie mit kardiovaskulär wirksamen Medikamenten nach kardiovaskulärem Risiko mit Ziel HbA1c < 7 %, aber auch nach Alter durchgeführt werden [9, 31]. Die interdisziplinäre Zusammenarbeit zwischen Urologen/Onkologen und Kardiologen ist hierbei hilfreich. Spezielle kardioonkologische Ambulanzen bzw. Schwerpunkte meist an großen universitären kardiologischen Einrichtungen wenden spezielle Algorithmen an, um so die bestmögliche onkologische als auch kardiovaskuläre Versorgung dieser Patienten zu ermöglichen. Eine Anbindung an solche kardioonkologischen Ambulanzen bzw. Schwerpunktkliniken ist daher mehr als wünschenswert – ein entsprechender Ausbau dieser Strukturen die notwendige Konsequenz [32].

Digitale Gesundheitsanwendungen (DiGA) in Form von Apps auf Rezept könnten künftig Arzt und Patienten unterstützen und das Bewusstsein für kardiovaskuläre Risiken bei PCa-Patienten weiter schärfen. So wurde beispielsweise interdisziplinär mit Urologen, PCa-Patienten und Psychologen das DiGA-Projekt PROSTANA® (FERRING Arzneimittel GmbH, Kiel, Deutschland) entwickelt [4]. Hier können aktuelle Informationen zu Erkrankung, Diagnose und Therapieoptionen auch unter Berücksichtigung eventueller kardiovaskulärer Risiken bzw. Vorerkrankungen abgefragt werden.

Die wachsende Zahl wissenschaftlicher Publikationen sowie neuer präklinischer und klinischer Studien zeigt, dass kardiovaskuläre Ereignisse bei PCa-Patienten zunehmend als Problem begriffen werden. Noch mehr als bisher sollten wir diese auch im Behandlungsalltag berücksichtigen. Dabei sollte im Sinne einer Vermeidung kardiovaskulärer Ereignisse die genaue Prüfung der Indikation für eine ADT an erster Stelle stehen.

## Fazit für die Praxis

- Kardiovaskuläre Ereignisse sind relevante Komplikationen bei PCa-Patienten (Prostatakarzinom), insbesondere unter Androgendeprivationstherapie (ADT) und neuen Androgenrezeptor- (AR-)gerichteten Medikamenten.
- Das Screening auf vorbestehende kardiovaskuläre Erkrankungen ist für die Risikominimierung bei Patienten mit PCa unerlässlich.
- Randomisierte klinische Studien, Metaanalysen und Daten aus dem Behandlungsalltag zeigen einen Vorteil von Gonadotropin-Releasing-Hormon-(GnRH-)Antagonisten im Vergleich zu GnRH-Agonisten bezüglich kardiovaskulärer Komplikationen.
- Eine umfassende Aufklärung sowie ein regelmäßiges Monitoring bezüglich kardiovaskulärer Ereignisse sollten fest in den Behandlungsalltag integriert werden.
- Bei notwendigen medikamentösen Interventionen ist eine enge Kooperation mit Kardiologen insbesondere bei schwerwiegenden oder komplexen Fällen im Rahmen kardioonkologischer Ambulanzen oder Spezialkliniken unerlässlich. Mögliche Medikamenteninteraktionen müssen bedacht werden.
